# Understanding Public Perceptions of Per- and Polyfluoroalkyl Substances: Infodemiology Study of Social Media

**DOI:** 10.2196/25614

**Published:** 2022-03-11

**Authors:** Hao Tian, Christy Gaines, Lori Launi, Ana Pomales, Germaine Vazquez, Amanda Goharian, Bradley Goodnight, Erica Haney, Christopher M Reh, Rachel D Rogers

**Affiliations:** 1 Office of Director National Center for Environmental Health Centers for Disease Control and Prevention Atlanta, GA United States; 2 Office of Associated Director Agency for Toxic Substances and Disease Registry Atlanta, GA United States; 3 Office of Community Health Hazard Assessment Agency for Toxic Substances and Disease Registry Atlanta, GA United States; 4 Guidehouse LLP Atlanta, GA United States

**Keywords:** PFAS, per- and polyfluoroalkyl substances, social media, public perceptions

## Abstract

**Background:**

Per- and polyfluoroalkyl substances (PFAS) are environmental contaminants that have received significant public attention. PFAS are a large group of human-made chemicals that have been used in industry and consumer products worldwide since the 1950s. Human exposure to PFAS is a growing public health concern. Studies suggest that exposure to PFAS may increase the risk of some cancers and have negative health impacts on the endocrine, metabolic, and immune systems. Federal and state health partners are investigating the exposure to and possible health effects associated with PFAS. Government agencies can observe social media discourse on PFAS to better understand public concerns and develop targeted communication and outreach efforts.

**Objective:**

The primary objective of this study is to understand how social media is used to share, disseminate, and engage in public discussions of PFAS-related information in the United States.

**Methods:**

We investigated PFAS-related content across 2 social media platforms between May 1, 2017, and April 30, 2019, to identify how social media is used in the United States to seek and disseminate PFAS-related information. Our key variable of interest was posts that mentioned “PFAS,” “PFOA,” “PFOS,” and their hashtag variations across social media platforms. Additional variables included post type, time, PFAS event, and geographic location. We examined term use and post type differences across platforms. We used descriptive statistics and regression analysis to assess the incidence of PFAS discussions and to identify the date, event, and geographic patterns. We qualitatively analyzed social media content to determine the most prevalent themes discussed on social media platforms.

**Results:**

Our analysis revealed that Twitter had a significantly greater volume of PFAS-related posts compared with Reddit (98,264 vs 3126 posts). PFAS-related social media posts increased by 670% over 2 years, indicating a marked increase in social media users’ interest in and awareness of PFAS. Active engagement varied across platforms, with Reddit posts demonstrating more in-depth discussions compared with passive likes and reposts among Twitter users. Spikes in PFAS discussions were evident and connected to the discovery of contamination events, media coverage, and scientific publications. Thematic analysis revealed that social media users see PFAS as a significant public health concern and seek a trusted source of information about PFAS-related public health efforts.

**Conclusions:**

The analysis identified a prevalent theme—on social media, PFAS are perceived as an immediate public health concern, which demonstrates a growing sense of urgency to understand this emerging contaminant and its potential health impacts. Government agencies can continue using social media research to better understand the changing community sentiment on PFAS and disseminate targeted information and then use social media as a forum for dispelling misinformation, communicating scientific findings, and providing resources for relevant public health services.

## Introduction

### Background

Per- and polyfluoroalkyl substances (PFAS) are a large class of manufactured chemicals that have been widely produced and used in industry and consumer products such as nonstick cookware, water-repellent clothing, stain-resistant fabrics, carpets, and other items since the mid-20th century [1-3]. The most commonly studied PFAS are perfluorooctanoic acid (PFOA) and perfluorooctane sulfonic acid (PFOS) followed by perfluorohexane sulfonic acid and perfluorononanoic acid. Chemical companies and other industry manufacturers began phasing out the use and manufacture of the PFOA and PFOS variants in the early 2000s [2,3]. However, PFAS persist in the environment, especially in certain foods, water sources, people, and wildlife worldwide owing to their long biological half-lives [4]. Some communities nationwide have been and likely will continue to be exposed to drinking water contaminated with PFAS owing to both new and historical exposure [4-7]. Research examining the impact of PFAS has varied; however, some studies indicate that exposure to certain PFAS may increase the risk of some cancers and have negative health impacts on the endocrine, metabolic, and immune systems [3,5-7]. In recent years, PFAS as environmental contaminants have received significant public attention owing to emerging evidence of widespread PFAS contamination, the development of new state and federal drinking water PFAS guidelines, and a handful of newly released PFAS-focused documentaries and news stories [8-13].

One way to assess and understand the increase in public attention to PFAS is to investigate PFAS-related activity on various social media platforms. Social media is a broad term used to describe web-based platforms that allow individuals as well as representatives of institutions (such as news media, government agencies, nongovernmental organizations, and advocacy groups) to publish content and connect with other users. Widespread adoption of social media apps has fundamentally changed the way the public disseminates and shares information [14]. In the past decade, social media platforms have been used to monitor disease outbreak patterns and communicate information to the public during emergency responses for bubonic plague, swine flu, seasonal influenza, and West Nile virus disease [15-21]. As today’s digital activity increases, the use of social media platforms to find and share information about public health issues continues to grow.

Some studies have explored the use of social media to communicate information about emerging health concerns such as the health effects of e-cigarettes [19-22]. A recent study [21] observed that Reddit posts included an abundance of requests looking for healthy alternatives to e-cigarettes and that Twitter posts tended to focus on information seeking related to regulations and policy debates around e-cigarettes. Variation in information seeking and discussion by platform suggests that some platforms may be more successful than others for government agencies in the reach and delivery of public health information and interventions. Therefore, it is important to analyze data from different social media platforms to gain a better understanding of social media users’ perceptions of PFAS.

### Objectives

Although we are not aware of any studies investigating PFAS-related social media activity, we believe that observing American social media users’ attitude toward PFAS across various platforms has the potential to help government agencies better understand public concerns, prevent and address the spread of misinformation, respond to new PFAS-related incidents, and develop targeted communication and outreach efforts, as demonstrated in other studies targeting different health issues [23,24]. This social media research aims to increase understanding of the public’s perception of PFAS and inform how best to reach affected communities with the appropriate health information and environmental health prevention services. The public receives health information from various sources and stakeholders. It is important to understand how perceptions of and dialogue about those sources may affect government agencies’ ability to effectively communicate health information. By conducting social media research and analysis of PFAS-related posts, government agencies and other partner organizations may become better equipped to respond to public concerns, questions, and requests.

This study has three objectives: (1) to understand how social media is used to share, disseminate, and engage in public discussions of PFAS-related information in the United States; (2) to identify common themes within PFAS-related mentions across social media platforms; and (3) to identify how social media engagement relates to various news events to better anticipate when and where to target outreach efforts using social media.

## Methods

### Data Collection

We first selected seven social media platforms—Facebook, Twitter, Reddit, YouTube, Nextdoor, Imgur, and Pinterest—that are likely used by the public to share environmental health–related information and investigated the availability and quality of data from these platforms across a recent 2-year window from May 1, 2017, to April 30, 2019. We focused our analysis on a 2-year window based on 2 primary factors. The first is an increase in media attention to PFAS in late 2017. The second is the dynamic and fluid nature of social media environments and the internet more broadly. Accordingly, we focused on a recent 24-month period that would enable sufficient time to identify patterns. Owing to the identified challenges in the collection, processing, and comparison of the data associated with these disparate social media platforms, we tailored our data collection to focus on Twitter and Reddit, the 2 platforms that provided access to data and metadata, allowing us to achieve the study objectives. We extracted the data on August 7, 2019, for Twitter (N=98,264) and Reddit (n=3126) through the use of NetBase (NetBase Solutions Inc), a third-party software vendor that facilitated access to the platforms’ full-stream application programing interfaces (eg, *firehose* application programing interfaces), which included all publicly available content (ie, not content from private accounts) featuring our identified key terms defined below within our specified time frame. On the basis of the scope of our research questions, we did not collect PFAS-related content posted from countries other than the United States.

### Ethics Approval

The data used for this study were based on publicly available information only and, thus, the study was determined to be exempt from the Centers for Disease Control and Prevention Institutional Review Board approval.

### Variables of Interest

Our key variable of interest was posts on *PFAS* or common alternatives, including *PFOA*, *PFOS*, and the hashtag derivatives of each term *(#PFAS*, *#PFOA*, and *#PFOS*) across social media platforms. These key term variables were selected based on their more prominent volume and use as identified in an initial exploration of PFAS-related key term frequency during the same time frame on Google Trends (data accessed on June 19, 2019).

Posts were classified as either original posts or engagement posts. Original posts were defined as first-instance comments. Engagement posts were secondary posts in the form of a reply or comment (including retweets on Twitter) to an original post. We acknowledge that these types of posts are different; however, we chose to aggregate at the *engagement* level so that engagement could be compared across platforms. On the basis of the study goals, additional variables of interest included post type, Twitter *likes*, date of post, and PFAS event or geographic location of social media user. Below are the key variables and their associated definitions.

- PFAS post (binary): a post, tweet, or comment on social media platforms that uses one or more of the terms PFAS,PFOA, or PFOS
- Post type (binary):original posts refers to first-time comments.Engagement posts refers to any reply, comment, or retweet that responds to or engages with an existing comment. The engagement ratio is the proportion of engagement posts to original posts. Engagement posts are defined similarly across different social media platforms, although they may be referred to with platform-specific language (eg, a retweet on Twitter)
- Likes (count): specific to Twitter data,likes indicates the number of Twitter users that clicked on like on a tweet
- Date (continuous): the day, month, or year of a tweet, post, or comment
- Geography (categorical): the national jurisdiction location of a PFAS-related post

Although original posts measure message reach, engagement posts measure interactive dialogue among social media users. Engagement ratio is defined as the proportion of engagement posts to original posts, which is an indicator of the degree of engagement by users via replies to, comments on, or retweets of an original post. For example, an engagement ratio of 2 suggests that users were 2 times more likely to reply to, comment on, or retweet an existing post than to generate an original post.

We manually reviewed 3 random samples (n=500) of the data sets to identify keywords or phrases for cleaning irrelevant or misleading data. This manual review was used to implement rule-based removal of similar irrelevant or misleading content throughout both data sets. Irrelevant data accounted for <1% of Twitter (254/98,264, 0.26%) and Reddit (18/3126, 0.58%) data. Examples of removed content included advertisements for various cookware products with marketing language such as *PFOA-free* and irrelevant posts of *PFAs*, an acronym for *protection from abuse* referencing an unrelated domestic violence abuse order.

### Quantitative Analysis

We analyzed and identified unique attributes of public engagement across each platform. We used descriptive statistics, including counts and frequencies of the key quantitative variables, to assess the incidence of PFAS discussions and to identify time, geographic, and entity patterns. We explored PFAS-related posts over time to identify spikes and assess their association with PFAS-related events. We obtained maps of tweets and Reddit posts by geographic area using a data visualization package (ggplot2) in the statistical software R (R Foundation for Statistical Computing) [25].

The purpose of the geographic analysis was to demonstrate which states had the highest number of PFAS-related posts broken down by original and engagement posts, count percentage, and the engagement to original post ratio for each state. Understanding geographic patterns in PFAS-related posts more broadly can help government agencies develop future outreach efforts.

That said, geographic metadata are not always provided by a given social media platform. For this analysis, we identified geographic information for tweets based on state-level geographic data available in an individual user’s profile. However, Reddit data did not provide geographic metadata for users’ posts. To approximate geographic concentrations of Reddit data, we built a complementary proxy using text analysis of geographic subreddit identification (eg, *r/Michigan*) and user mentions of specific jurisdictions. To complement geographic approximation, we also conducted a scan of post titles that directly referenced a specific geographic location. Although not providing an exact comparison, geographic analysis through these 2 approaches provided insight into potentially underlying differences in PFAS conversations by geography. To account for population variance across states, we normalized the data using 2010 census data to provide a proportional jurisdiction ratio of per 100,000. Our dissimilar approach to obtaining geographic information, coupled with variance in sample sizes between the 2 platforms, is likely to influence geographical findings and related conclusions.

### Qualitative Analysis

We used the constant comparison method based on grounded theory [26,27] to inductively analyze posts and comments, coupled with deductive analysis to identify and analyze major discussion themes on social media platforms. A total of 3 people used Microsoft Excel to individually open code 3 subsets of randomly sampled text (1500 in total; 500/1500, 33.33% per subset) for common themes, cross-validation, and collective refinement of themes. Through an iterative process, some coding categories were collapsed into larger concepts until no further themes emerged in the subsequent analysis, suggesting that we had reached saturation of themes. The team subsequently developed search criteria with a unique set of keywords for each theme based on the initial open coding process (see the *Thematic Analysis* section for example keywords for each theme). Themes were not mutually exclusive and, therefore, each unit of analysis had the potential to be counted across multiple themes.

### Mixed Methods Analysis

As a follow-up to the qualitative analysis, we conducted a series of regression analyses to test whether posts containing words from the search criteria established in the qualitative analysis differed from one another and from posts that did not use the search criteria in terms of their level of engagement on the Twitter and Reddit platforms. Search criteria were used to separate posts into thematic groups, and the relationship between these nonexclusive groups was established on two indices measuring user engagement: categorization as a reply to an original post and number of *likes* on Twitter. The data set used for comparison included only social media users of the Twitter and Reddit platforms. The level of analysis was posts and comments, and the unit of analysis was comment or post and not individual users.

The analyses consisted of a logistic regression to examine the effect of the 3 thematic groups on a binary outcome of original posts versus engagement posts. In other words, the logistic regression analysis compared each of the 3 themes to one another in terms of the relative quantity of engagement posts to original posts (ie, level of engagement). In addition, we conducted a Poisson regression analysis to look at the effect of the same predictors on a count outcome representing the number of *likes* that a tweet received on Twitter, a secondary indicator of user engagement. As *likes* is a metric that is unique to Twitter and the sample of Reddit data was much smaller than the Twitter sample, these follow-up mixed methods analyses were limited to the Twitter data. We were unable to conduct the same analyses among Reddit data as we did not have access to upvote or downvote information, a measure used to demonstrate that a user likes or dislikes a post. Furthermore, the smaller number of posts for Reddit provided much lower statistical power than the Twitter data, and the limitation for post length for Twitter and not for Reddit limited our ability to compare the 2 platforms.

## Results

### Platform Volume, Post Type, and Term Use

As seen in Table 1, there were a total of 101,390 PFAS-related posts on Twitter and Reddit during the 2-year window. Twitter had a significantly greater volume of PFAS-related posts compared with Reddit (98,264 vs 3126).

**Figure 1:**
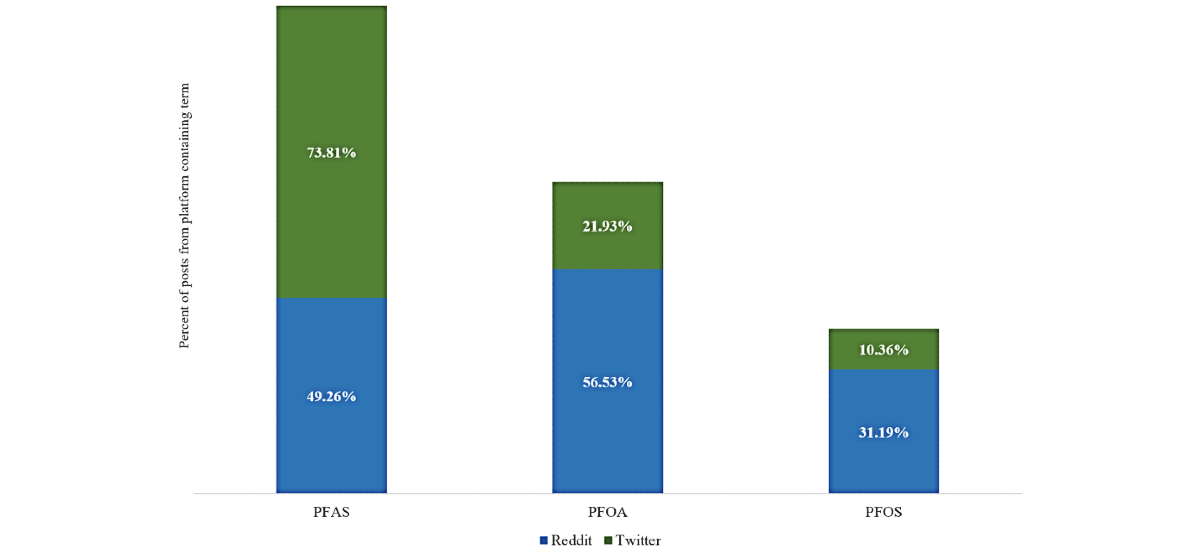
Key term distribution across all posts by social media platform (Twitter: N=98,264; Reddit: N=3126). PFAS: per- and polyfluoroalkyl substance; PFOA: perfluorooctanoic acid; PFOS: perfluorooctane sulfonic acid.

Understanding PFAS-related term use clarifies how social media users were interfacing with PFAS-related conversations on social media. Among Twitter data, social media users used the term *PFAS* in 73.81% (72,528/98,264) of all posts compared with *PFOA* (21,545/98,264, 21.93%) and *PFOS* (10,183/98,264, 10.36%). Given that a post may include multiple terms, the aggregate total resulted in a value of >100%. We saw a different pattern of term use among Reddit posts whereby users most mentioned the term *PFOA* (1767/3126, 56.53%) followed by *PFAS* (1540/3126, 49.26%) and *PFOS* (974/3126, 31.16%; Figure 1).

**Table 1:** Counts of observations by social media platform.

		Original posts, n (%)	Engagement posts^a^, n (%)	Total, n (%)
**Platform**				
	Twitter (tweets)	36,795 (37.44)	61,469 (62.55)	98,264 (100)
	Reddit (posts)	424 (13.56)	2702 (86.44)	3126 (100)
Total		37,219 (36.71)	64,171 (63.29)	101,390 (100)

^a^Engagement posts include all replies and retweets.

### Timing and Event Analysis

#### Overview

**Figure 2:**
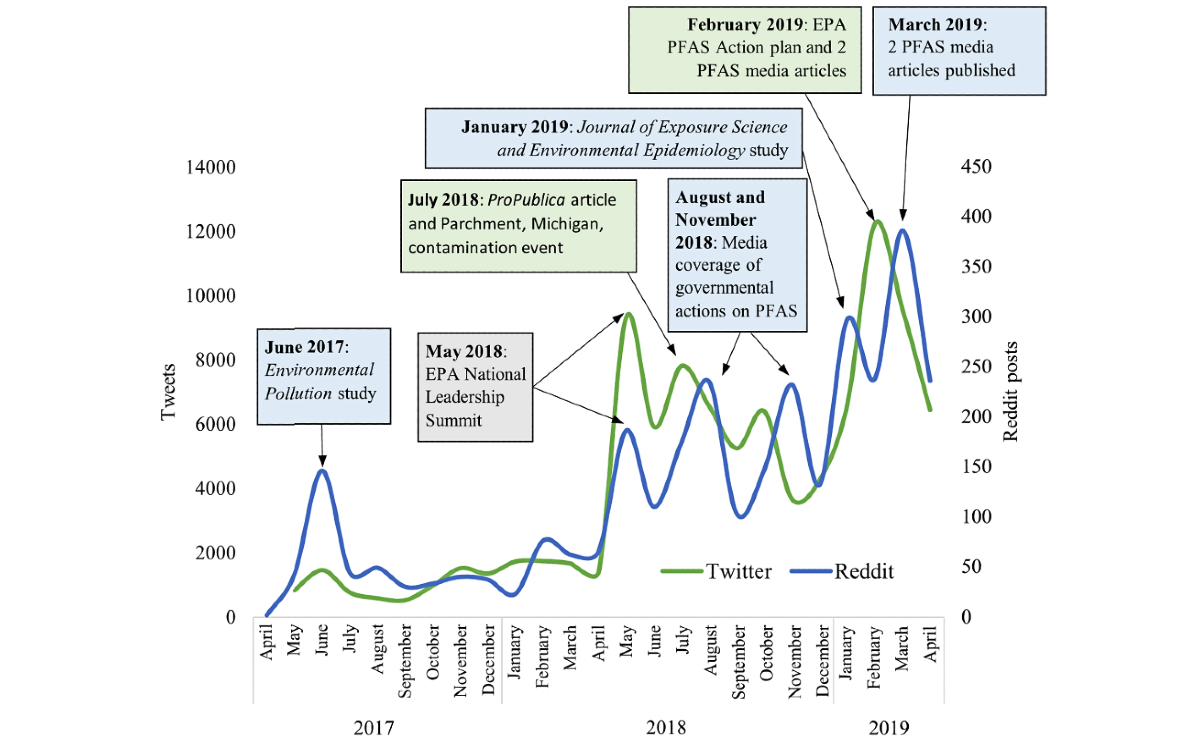
Number of per- and polyfluoroalkyl substance (PFAS)-related Twitter and Reddit posts by date with annotated events. EPA: Environmental Protection Agency.

The volume of posts over time underscores a collective rise in interest in and awareness of PFAS. Driving this increase were 9 notable spikes in the volume of public discussion of PFAS (3 on Twitter and 6 on Reddit). We evaluated the data retrospectively and conducted text analysis to determine the primary PFAS-related topics and events mentioned in the posts associated with the spikes (Figure 2).

#### Twitter Spikes and Events

The 3 primary spikes in PFAS-related discussions on Twitter occurred in May 2018, July 2018, and February 2019. The content analysis of posts during these periods revealed that the spike in May 2018 was attributed to the high volume of posts about the US Environmental Protection Agency (EPA) National Leadership Summit (which happened in May 2018). In July 2018, a smaller but notable spike occurred that was attributed to discussions of a *ProPublica* article on government responses to PFAS [28] and a local news article about the PFAS water contamination in Parchment, Michigan [29]. Finally, the spike in February 2019 corresponded with the release of a PFAS action plan by the EPA and greater national-level reporting on PFAS by mainstream publications, such as *The New York Times* articles on PFAS exposure in military families [30] and government response [31]. In several instances, spikes occurred simultaneously or were slightly delayed across platforms, indicating the potential for a sustained discussion over time and across platforms of critical PFAS events.

#### Reddit Spikes and Events

The 6 primary spikes in PFAS-related discussions on Reddit occurred in June 2017, May 2018, August 2018, November 2018, January 2019, and March 2019. The June 2017 spike was primarily related to the publication and release of a peer-reviewed study in *Environmental Pollution* on PFAS exposure in the mid–Ohio River Valley from 1991 to 2012 [32]. The spike in May 2018 was attributed to the high volume of posts about the US EPA National Leadership Summit. A noticeable spike in Reddit activity in August 2018 was related to the publication of 2 different media articles. One was from *ProPublica* on government response [28], the same article found in the Twitter July 2018 spike. The other article was from *MLive*, a local Michigan news site, focused on governmental action toward PFAS contamination [5]. The reprinting of the *ProPublica* article [28] by CNBC on November 12, 2018, led to an increase in Reddit activity, with numerous users posting links to and comments about the article within the subreddit *r/news*. Another noteworthy increase in PFAS-related Reddit activity was largely in response to a peer-reviewed study published in January 2019 in the *Journal of Exposure Science and Environmental Epidemiology* on serum concentrations of PFAS and exposure-related behaviors in African American and non-Hispanic White women [33]. Finally, the March 2019 spike was associated with 2 media articles on the regulatory process of the EPA and PFAS exposure in military families from *The Guardian* [34] and *The New York Times* [30], respectively.

### Geographic Analysis

#### Twitter Geographic Analysis

**Figure 3:**
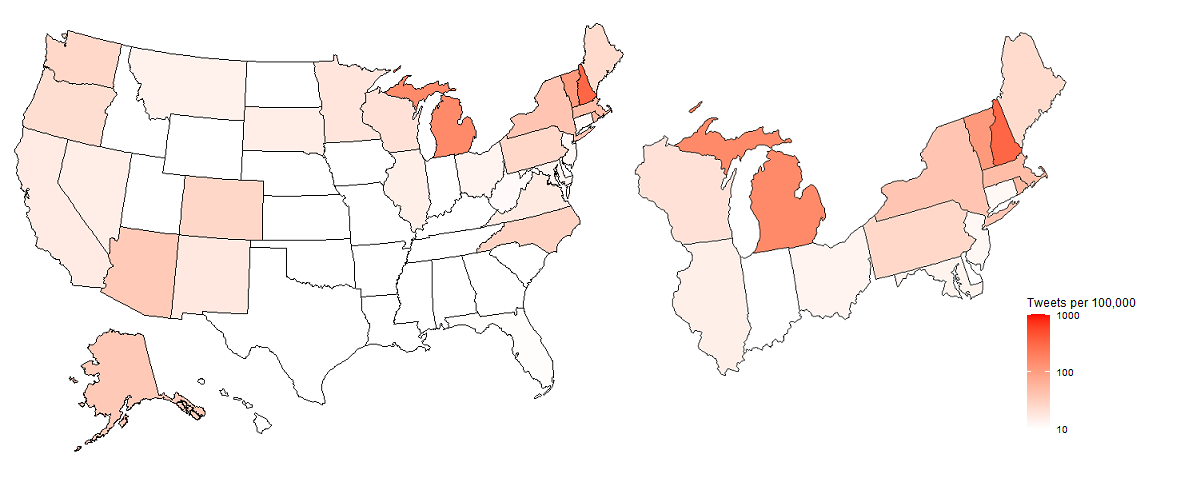
Choropleth of tweets per 100,000 people by state.

We examined the pure volume of tweets, including both engagement and original, irrespective of state population, revealing that 15.83% (15,560/98,264) of the tweets occurred in Michigan followed by New York (7765/98,264, 7.9%), District of Columbia (DC; 6822/98,264, 6.94%), California (6075/98,264, 6.18%), and New Hampshire (4262/98,264, 4.34%). However, this measure of pure tweet volume does not account for differences in population sizes across jurisdictions. Figure 3 incorporates data from the 2010 US census to provide a normalized comparison of tweets per 100,000 people by state. Once normalized, DC rose to 1133 tweets per 100,000, nearly 4 times that of New Hampshire with 323 tweets per 100,000. The remaining three top-ranking states were Michigan, Vermont, and Rhode Island with 157, 112, and 56 tweets per 100,000, respectively.

Table 2 highlights the engagement ratio for the top 5 jurisdictions with the highest number of tweets per 100,000 population. Among the 5 jurisdictions with the highest PFAS-related Twitter volume per 100,000 population, New Hampshire displayed the highest engagement ratio (2.71) followed by Rhode Island (2.52), DC (1.23), Michigan (1.2), and Vermont (0.9).

**Table 2:** Top 5 jurisdictions with the highest number of tweets per 100,000 population and engagement ratio.

Jurisdiction	Tweets per 100,000 population, N	Original posts, N	Engagement posts, N	Engagement ratio
District of Columbia	1133.74	3057	3765	1.23
New Hampshire	323.74	1149	3113	2.71
Michigan	157.43	7059	8501	1.2
Vermont	112.99	372	335	0.9
Rhode Island	56.15	168	423	2.52

#### Reddit Geographic Analysis

**Figure 4:**
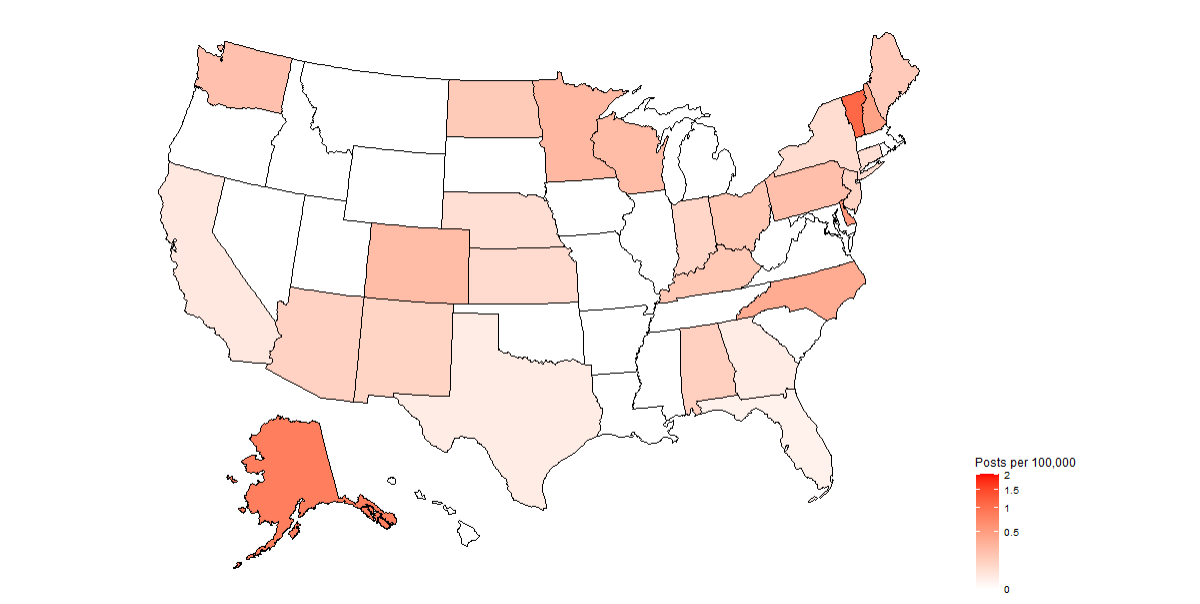
Choropleth of Reddit posts per 100,000 people by state.

Of the 3126 posts in the Reddit data set, 443 (14.17%) contained jurisdictional information, allowing for geographic analysis. The remaining 85.83% (2683/3126) did not have any information for geographic linking. The top five states for PFAS-related posts by pure volume, including both original and engagement posts, were Michigan (206/443, 46.5%), North Carolina (35/443, 7.9%), Pennsylvania (28/443, 6.3%), Ohio (19/443, 4.3%), and Minnesota (14/443, 3.2%). The top jurisdictions by Reddit posts per 100,000 population were Michigan (2.08), Vermont (1.12), Alaska (0.84), Delaware (0.56), New Hampshire (0.46), North Carolina (0.37), Minnesota (0.26), and Wisconsin (0.25; Figure 4).

Table 3 highlights the engagement ratio for the top jurisdictions with the highest number of Reddit posts per 100,000 population. Minnesota displayed the highest engagement ratio (6) followed by North Carolina (4), Michigan (3.29), Vermont (2.50), and Wisconsin (1.80). The engagement ratios for Alaska, Delaware, and New Hampshire could not be computed (*N/A* in Table 3) as the number of original posts was 0 for these states.

**Table 3:** States with the highest number of Reddit posts per 100,000 population and engagement ratio.

Jurisdiction	Reddit posts per 100,000 population	Original posts, N	Engagement posts, N	Engagement ratio
Michigan	2.08	48	158	3.29
Vermont	1.12	2	5	2.50
Alaska	0.84	0	6	N/A^a^
Delaware	0.56	0	5	N/A
New Hampshire	0.46	0	6	N/A
North Carolina	0.37	7	28	4
Minnesota	0.26	2	12	6
Wisconsin	0.25	5	9	1.80

^a^N/A: not applicable.

### Thematic Analysis

We identified the following three topics as the most prevalent themes, ordered from most to least commonly mentioned: (1) social media users discuss PFAS as an immediate public health concern, (2) social media users desire clarity on government agencies’ roles related to PFAS, and (3) social media users discuss general mistrust of PFAS-related information.

#### Social Media Users Discuss PFAS as an Immediate Public Health Concern

We defined this theme, hereafter abbreviated as *PFAS are a health concern*, by the volume of posts discussing the health impacts of PFAS. Some of the thematic excerpts included a discussion of the products known to contain a PFAS derivative and their associated health effects. Examples of keywords associated with this theme included *hazard*, *crisis*, *threat*, *exposure*, *dangerous*, *unsafe*, and *risk*. Many social media users believe that PFAS are dangerous human-made chemicals that contribute to wider environmental and health issues. A significant portion of the posts associated with this theme included dialogue on the discovery of new PFAS contamination sites across the country, *breaking* news articles or studies reporting new locations with high concentrations of PFAS, and concerns for potential adverse health effects from personal exposure to PFAS. In addition, social media posts containing this theme often implicitly or explicitly expressed a lack of clarity on PFAS health effects or uncertainty on where to access relevant health information. Discussions often included questions for clarification or requested links to PFAS-related information.

#### Social Media Users Desire Clarity on Government Agencies’ Roles Related to PFAS

We defined this theme, hereafter abbreviated as *clarity on government agencies’ roles*, by the volume of posts mentioning government agencies (eg, state and local health government and federal agencies) and discussing the need for government leadership for PFAS-related community outreach and dissemination of standardized information. Examples of keywords associated with this theme included *enforce*, *state*, *regulate*, *mitigate*, and *protect*. Social media users largely engaged with PFAS discussions as it related to a desire for more government participation at the local, state, and federal levels. Users frequently connected PFAS-related issues and concerns to contaminated water and drinking water supplies and called on the government for more active support in producing evidence-based science to identify and protect the public from further PFAS exposure. The public’s confusion and uncertainty on various agencies’ roles in PFAS-related activities provides an opportunity for government agencies to ensure their messaging outlines how the actions they are taking will protect public health.

#### Social Media Users Discuss General Mistrust of PFAS-Related Information

We defined this theme, hereafter abbreviated as *mistrust of PFAS information*, by the volume of posts discussing mistrust of the available information related to PFAS. Examples of keywords associated with this theme included *corrupt*, *greed*, *litigation*, and *accountable*. In the last decade, there has been an exponential increase in scientific literature on the health effect of PFAS and many ongoing changes in PFAS information, in part because of jurisdictional attempts and successes in changing health exposure thresholds [3,5,10]. The data suggest that many social media users felt unable to determine the credibility of certain sources of PFAS information, particularly when conflicting information is presented by media outlets and various sources. Many users commented on the potential for political bias in journalism, often looking for authoritative sources of scientific information. Social media users often sought to expand their knowledge by discussing PFAS-related information with other users and expressed an overarching frustration based on the need to piece together accurate PFAS information.

#### Twitter Thematic Analysis

**Figure 5:**
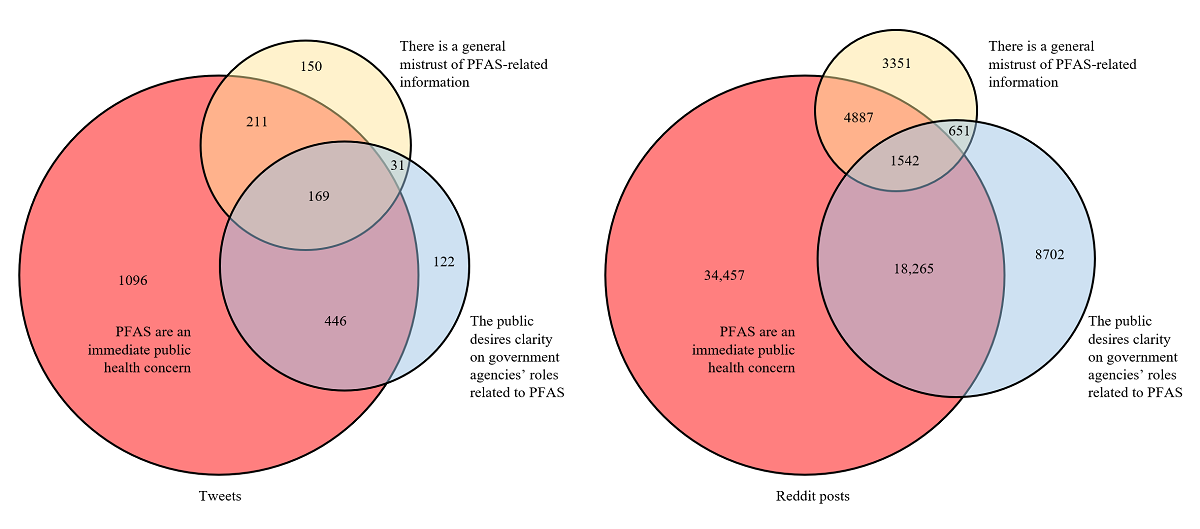
Overlap of tweets or Reddit posts containing at least one thematic code (Twitter: N=98,264; Reddit: N=3126). PFAS: per- and polyfluoroalkyl substance.

Of the 98,264 tweets, 59,151 (60.2%) received the *PFAS are a health concern* code, 29,160 (29.68%) received the *clarity on government agencies’ roles* code, and 10,431 (10.62%) received the *mistrust of PFAS information* code. Of the 98,264 total tweets, 71,855 (73.12%) received *at least* one thematic code, and the remaining 26,409 (26.88%) received no code. As seen in Figure 5, approximately 47.33% (46,510/98,264) received exactly 1 code, and 24.22% (23,803/98,264) received 2 codes. Approximately 1.57% (1542/98,264) received all 3 codes. See the *Thematic Analysis* section above for clarification of theme definitions.

#### Reddit Thematic Analysis

Of the 3126 posts in the Reddit data set, 1922 (61.48%) received the *PFAS are a health concern* code, 768 (24.57%) received the *clarity on government agencies’ roles* code, and 561 (17.95%) received the *mistrust of PFAS information* code. Of the complete Reddit data set of 3126 posts and comments, 2225 (71.18%) received at least one code, and 901 (28.82%) received no code. See the *Thematic Analysis* section above for clarification of theme definitions.

As shown in Figure 5, approximately 43.76% (1368/3126) received exactly 1 code, and 22% (688/3126) received 2 codes. Approximately 5.41% (169/3126) received exactly 3 codes.

#### Mixed Methods Analysis

Posts that received the *PFAS are a health concern* code were 1.59 times as likely as other PFAS-related posts to be categorized as engagement (ie, a reply, comment, or retweet that responded to or engaged with an existing comment) rather than volume (ie, an original post that did not reply, comment, or retweet a previously existing comment). Posts that received the *clarity on government agencies’ roles* code (odds ratio 1.35, 95% CI 1.31-1.39) and *mistrust of PFAS information* (odds ratio 1.36, 95% CI 1.3-1.42) code were also significantly more likely to be categorized as engagement rather than volume (Table 4).

Post hoc tests showed that posts related to the theme *PFAS are a health concern* were significantly more likely to be labeled as engagement posts compared with posts categorized into the other 2 themes. However, the themes *clarity on government agencies’ roles* and *mistrust of PFAS information* were statistically equal in their relative levels of engagement.

The number of *likes* for a post ranged from 0 to 1447 within the data set. As shown in Table 5, a post categorized as *PFAS are a health concern* was 1.78 times as likely to receive *likes* compared with other PFAS tweets. Tweets categorized as *mistrust of PFAS information* were 1.35 times as likely to receive *likes*. However, the *clarity on government agencies’ roles* theme received 94% as many *likes* (incident rate ratio=0.94; *P*=.001) when compared with other PFAS-related tweets. Furthermore, when conducting 2-way post hoc comparisons across themes, we found that all themes were statistically significantly different from one another in terms of how often they received *likes* by Twitter users.

**Table 4:** Logistic regression results of engagement on thematic categories.

Variable	Values			
	Estimate (SE)	*Z*	*P* value	OR^a^ (95% CI)
Intercept	0.12 (0.01)	11.26	<.001	—^b^
PFAS^c^ are a health concern	0.46 (0.01)	34.11	<.001	1.59 (1.54-1.63)
Mistrust of PFAS information	0.31 (0.02)	13.65	<.001	1.36 (1.3-1.42)
Clarity on government agencies’ roles	0.3 (0.01)	20.12	<.001	1.35 (1.31-1.39)

^a^OR: odds ratio.

^b^Odds ratio not provided for intercept.

^c^PFAS: per- and polyfluoroalkyl substance.

**Table 5:** Poisson regression results of likes on thematic categories.

Variable	Values			
	Estimate (SE)	*Z*	*P* value	IRR^a^ (95% CI)
Intercept	−2.25 (0.02)	−135.40	<.001	0.11 (0.1-0.11)
PFAS^b^ are a health concern	0.57 (0.02)	31.09	<.001	1.78 (1.71-1.84)
Mistrust of PFAS information	0.30 (0.02)	12.74	<.001	1.35 (1.29-1.41)
Clarity on government agencies’ roles	−0.06 (0.02)	−3.27	.001	0.94 (0.91-0.98)

^a^IRR: incident rate ratio.

^b^PFAS: per- and polyfluoroalkyl substance.

## Discussion

### Social Media Data Usability for Public PFAS Interest Analysis

There were several challenges identified in the collection, processing, and comparison of data associated with the original 7 disparate social media platforms we sought to evaluate. Owing to data access restrictions on Nextdoor, we could not collect any PFAS-related mentions at all on the platform nor could we capture aggregated or descriptive statistics of PFAS mentions from the platform. On Pinterest, we ran into limitations collecting PFAS mentions with required metadata (eg, time and regions across the United States), which removed the data’s potential comparative value. Attempts at data collection on YouTube and Imgur did not reveal substantial user mentions of PFAS-related key terms during the period of analysis. The full sample from YouTube included <300 videos, and Imgur featured a single post.

Although Facebook can be a valuable data source for analyzing social media use, Facebook only enables data to be collected from preidentified public pages. Therefore, we determined that it was not appropriate to include Facebook data in this untargeted analysis that aimed to explore and analyze the general patterns and uses of social media for PFAS-related topics. Given these challenges, we focused our analyses on two platforms—Twitter and Reddit—based on similarities in metadata and relatively large sample sizes.

### Use Trends Across Social Media Platforms

Social media data provide a lens to understand perceptions about rising public health phenomena (see the *Limitations* section for further discussion of social media user population representativeness). Comparing the availability of PFAS-related data and metadata on different social media platforms and the Twitter and Reddit findings, our analysis indicates variation in post volume by platform. Our research over the 2-year period shows increasing public dialogue on PFAS. Although there are several other platforms that can contain meaningful insights into public perceptions of PFAS, for this analysis, Twitter and Reddit were the most appropriate for understanding geographic trends and theme sentiment. Although Facebook is the most widely used social media network [35] and can be a valuable data source for analyzing social media use, Facebook only enables data to be collected from preidentified public pages. Therefore, Facebook data can be suitable for a targeted research approach whereby researchers first select specific public Facebook pages associated with topics of interest and then collect data from those pages. This data retrieval restriction limits the use of Facebook for broad identification of related content across the entire platform and detecting general use trends.

The rising volume of posts across Twitter and Reddit and the growing visibility of PFAS across local and national news suggest an increasing interest in PFAS-related information. Considering each of the social media sources initially examined for this study, Twitter was the predominant platform for PFAS-related posts, likely owing in part to its ease of use in sharing ideas, real-time information, and trending news. However, despite having a relatively larger proportion of original posts, users engaged more frequently with Reddit posts, suggesting a more interactive platform. On the basis of our content analysis, Reddit users often provided their impressions, opinions, and perceptions of PFAS-related topics with other users. Individuals using Reddit to discuss PFAS tended to engage in follow-up discussions with the goal of gaining additional factual or anecdotal information or to provide additional resources. These differences in posts across social media platforms provide an initial understanding of the ways in which government agencies can further investigate PFAS sentiment among social media users. Through the themes that this research identified, our findings suggest that social media posts can help provide insight into some portions of the public’s accurate or inaccurate understanding of PFAS-related information and determine some gaps in public knowledge.

### Geographic and Event Trends

Understanding the prevalence of PFAS-related posts by jurisdiction provides a measure for PFAS public health professionals, organizations, and federal agencies to provide tailored intervention information, remediation, and community outreach by jurisdiction. The geographic data revealed a pattern of high-volume PFAS-related posts among jurisdictions affected by PFAS contamination during the time frame. Our analysis suggests that individuals living in jurisdictions with recent PFAS events appeared to be more engaged with web-based discussions that included continuous and active coverage of PFAS events by local news outlets. DC, New Hampshire, and Michigan were the top-ranked jurisdictions based on posts per 100,000 across both platforms. Much of the social media dialogue associated with Michigan and New Hampshire was related to PFAS contamination incidents or sites such as Parchment in Michigan and Pease International Tradeport in New Hampshire.

### Thematic Trends

Our analysis revealed that many posts described the contaminants as a public health threat and national crisis, especially among those living in PFAS-affected communities. As more individuals become aware of and are affected by PFAS, there is a growing sense of urgency to understand the health impacts and protective behaviors associated with PFAS exposure. This is exemplified through the predominant theme found in our analysis—*PFAS are a health concern*. Our findings suggest that both Twitter and Reddit were used to question, share, engage with, and react to evolving information related to discovered PFAS contamination. This is underscored by individual desires for access to concrete and reliable evidence-based information about PFAS and their impacts on human health and the environment.

Our results showed a notable overlap between *PFAS are a health concern* and *clarity on government agencies’ roles*. Some posts stressed the notion that scientific, academic, and environmental communities could play a positive and significant role in shaping future PFAS responses and interventions by acting both as scientific experts and trustworthy resources for answering outstanding questions on both health and environmental risks. These findings present an opportunity for government agencies to provide scientific information, answer questions, and correct misperceptions. American social media users are particularly interested in understanding what is being done to mitigate exposure, the potential human health effects, and obtaining information on tangible steps individuals can take to manage their own risk. Although there is still much to be known about PFAS, our findings suggest that there is an unmet need among the public for clarity on who is leading the charge in scientific discoveries, cleanup and protection, and dissemination of scientific health-related information. Given the core theme *clarity on government agencies’ roles*, government agencies have an opportunity to play a lead role in developing and distributing materials to the public on what is known about PFAS detection and to effectively communicate what remains unknown. This will not only provide PFAS information but also demonstrate the agencies’ roles as it relates to PFAS and health. Furthermore, by capitalizing on social media’s interactive format, government agencies can communicate information in these forums in a way that reflects audience preferences, stimulates conversation on relevant critical topics, and provides content that can be tailored to each platform’s application.

On the basis of our findings, individuals and communities negatively affected by PFAS feel uninformed and subsequently excluded from proposed or in-progress solutions. There is a strong desire on social media for a trusted source of PFAS-related health information on everything from breaking news and events to longer-term response plans.

Our analysis found that PFAS content is best created with a lens that acknowledges sensitivities within current public perceptions. It is preferred that outreach efforts and scientific findings ensure clarity in word choices and accurate framing of hot topics to mitigate disinformation and encourage social media users’ engagement. Dissemination strategies may be developed through partnerships with trusted scientific and academic organizations to dispel misinformation and demonstrate collaborative active leadership on the topic. In this way, continuous and ongoing evaluation of PFAS-related social media discussions may be advantageous, especially as new discussions arise across social media platforms and further communication materials are developed and deployed nationally. On social media platforms, government agencies can demonstrate real-time awareness of current PFAS-related issues and provide diverse content to address geographically specific concerns. By better understanding social media user interests, concerns, opinions, and perceptions of PFAS, environmental health professionals will be better equipped to uncover innovative ways to inform and engage citizens on important environmental health topics.

### Limitations

There are several limitations to our analysis. First, differences may exist between social media users and the general population [36], so our findings should not be extrapolated to the general population. Second, given the jurisdictional patterns found when sorting by our engagement ratio measure, we believe this measure has several inherent flaws. Most notably, it indicates instability among small population jurisdictions, as seen with several small states that were among the top 5 when measured by engagement ratio but not among the top 5 when measured by original post volume and original post volume by 100,000 people. In addition, our Reddit data were substantially truncated because of the lack of geographic indicators in the metadata. This resulted in a small sample size and more volatility in the geographic analysis across this platform. As events were determined post hoc, there was an inherent collinearity between the geography and event measures, resulting in potentially inflated SEs and very little improvement in the predictive value of the outcome. The location-masking tools and decisions to opt out of location information may also have had an impact on the geographic analysis. The length of posts allowed by Twitter also differed from what is allowed by Reddit, which limited our ability to compare results between platforms. Third, we must acknowledge the potential existence of social media *bots*—an autonomous account or network of accounts that posts and shares content according to specific predetermined rules—within the evaluated data sets that was not an immediate focus of this research effort but nevertheless may have had an impact on the spread of PFAS-related information [37]. Finally, data generated by social media platforms create unique research challenges, primarily as social media platforms create an exceptionally dynamic data environment. Specifically, across social media platforms, content is being created, disseminated, reacted to, and interacted with constantly and in real time. At any moment, a new or returning user can view a video, like a post, unlike a post, post a comment, or delete a previous comment. Thus, this dynamic communication environment presents challenges to those conducting research with its data as the data represent a finite snapshot in time rather than a continuous cumulative capture.

### Conclusions

On the basis of the analysis of social media posts and activity, we find that PFAS are perceived as an immediate public health concern and there is a growing sense of urgency to understand this emerging contaminant and its potential health impacts on social media. Twitter is the predominant platform among the 7 investigated social media platforms for PFAS-related posts based on the pure volume of key terms mentioned during the 2-year time frame of the analysis. However, despite Twitter having a relatively larger proportion of original posts, Reddit posts were more frequently engaged with by users, suggesting a more interactive rather than passive platform. Some social media users seek to understand long- and short-term health risks and access reliable PFAS information to make personal decisions that mitigate health risks. All of this underscores an opportunity for a robust public health response. Government agencies can continue using social media research to better understand the changing public sentiment on PFAS and the critical topics of interest among affected communities and then use social media as a forum for dispelling misinformation, communicating scientific findings, and providing resources for relevant public health services. Through these findings on geographic and event-based trends in PFAS-related discussions, government agencies and other partner organizations are better equipped to disseminate targeted PFAS-related scientific information and conduct community outreach.

## Abbreviations

DC: District of Columbia
EPA: Environmental Protection Agency
PFAS: per- and polyfluoroalkyl substance
PFOA: perfluorooctanoic acid
PFOS: perfluorooctane sulfonic acid
